# MRSA Harboring *mec*A Variant Gene *mec*C, France

**DOI:** 10.3201/eid1809.111920

**Published:** 2012-09

**Authors:** Frederic Laurent, Hubert Chardon, Marisa Haenni, Michele Bes, Marie-Elisabeth Reverdy, Jean-Yves Madec, Evelyne Lagier, François Vandenesch, Anne Tristan

**Affiliations:** French National Reference Centre for Staphylococci, Lyon, France (F. Laurent, M. Bes, M.-E. Reverdy, F. Vandenesch, A. Tristan);; Hospices Civils de Lyon, Lyon (F. Laurent, M. Bes, M.-E. Reverdy, F. Vandenesch, A. Tristan);; Aix-en-Provence Hospital, Aix-en-Provence, France (H. Chardon, E. Lagier);; and French Agency for Food, Environmental, and Occupational Health and Safety, Lyon (M. Haenni, J.-Y. Madec)

**Keywords:** methicillin-resistant Staphylococcus aureus, MRSA, bacteria, mecA, mecC, mecA_LGA251_, variant gene, humans, animals, antimicrobial drug resistance, France

## Abstract

We describe human cases and clustered animal cases of *mec*A_LGA251_–positive methicillin-resistant *Staphylococcus aureus* in France. Our report confirms that this new variant has a large distribution in Europe. It may represent a public health threat because phenotypic and genotypic tests seem unable to detect this new resistance mechanism.

Since its first description in the early 1960s, methicillin-resistant *Staphylococcus aureus* (MRSA) has become a major public health issue because of worldwide spread of several clones. More than 20 years later, the specific genetic mechanism of its resistance has been identified as a mobile genetic element (staphylococcal cassette chromosome *mec*) integrated into the *S. aureus* chromosome, within which the *mec*A gene encodes a specific methicillin-resistant transpeptidase (penicillin-binding protein 2a) [PBP2a] (*1*). This protein has a low affinity for β-lactam antimicrobial drugs. Thus, bacteria expressing this protein are resistant to all types of these drugs.

A new divergent *mec*A homolog (*mec*C or* mec*A_LGA251_, in reference to LGA251 isolates from which it was characterized) (*2*,*3*) was recently described in a novel staphylococcal cassette chromosome *mec* named type XI (*2*). This newly identified protein has <63% aa identity with PBP2a encoded by *mec*A and was described in *S. aureus* or coagulase-negative staphylococci. This new *mec*A homolog has been detected in bacteria from dairy cattle in England and humans in England, Scotland, and Denmark (*3*). We report the emergence of human and animal cases of *mec*A-variant MRSA identified in France.

## The Study

The first case was detected in a 67-year-old man admitted to Aix-en-Provence Hospital in southern France on November 8, 2007, because of suspected joint infection of his left knee 3 years after total knee joint replacement. He reported gonalgia that had been present for 6 months. The patient was apyretic but asthenic and reported a 10-kg weight loss. He had voluminous knee effusion. The synovial fluid leukocyte count was >1,000 cells/mm^3^ with 86% neutrophils, findings consistent with a prosthetic knee infection (*4*). The patient had a deep and hyperkeratosic lesion on his left heel that was considered to be the source of infection.

Direct microscopic examination of the fluid identified gram-positive cocci in grape-like clusters. *S*. *aureus* was identified in pure culture. Susceptibility tests performed by using the disk diffusion method as recommended by the Société Française de Microbiologie (Paris, France) (www.sfm-microbiologie.fr) showed methicillin resistance. The presence of the *mec*A gene was investigated by using 2 methods, an-in house PCR (*5*) and the GenoType *Staphylococcus* test (Hain Lifescience GmbH, Nehren, Germany), but results were negative.

A 2-stage revision of arthroplasty of the infected knee was performed. Initial treatment was rifampin (1,800 mg/day) and ofloxacin (400 mg/day) for 5 months. Ofloxacin was then replaced with clindamycin (1,800 mg/day) for 2 months because of quinolone-induced tendinitis. The unusual length of treatment was required because of an abnormal delay in healing, persistence of knee inflammation, and subclinical inflammatory syndrome. The clinical outcome was favorable at a 3-year follow-up.

The second case was detected in 2 cows with clinical mastitis on the same farm in the Meurthe-et-Moselle District of northeastern France in December 2008. Two *S. aureus* strains were isolated from milk samples from these 2 cows. Drug susceptibility profiles determined by using the disk diffusion method were identical; both isolates showed methicillin resistance and susceptibility to other antimicrobial drugs, an unusual profile in veterinary microbiology. Only 1 strain was stored and sent to the French Agency for Food Environmental and Occupational Health and Safety (Lyon, France) where a *mec*A PCR (*6*) result was negative.

After failure of empiric treatment with cefalonium, a first-generation cephalosporin, the 2 cows were successfully treated with neomycin/spiramycin. Because these mastitis cases occurred in a context of recurrent bacterial infections (clinical and subclinical mastitis caused by *S. aureus* in dairy cows, diarrhea caused by *Escherichia coli* in veal calves), hygienic measures were instituted, including decontamination of milking machines, disinfection of teats before milking, and application of standard practices for infection control. These measures were successful, and MRSA was not detected again on this farm.

Using primers and the protocol reported by García-Álvarez et al. (*3*), we detected a *mec*A variant (*mec*A_LGA251_) in both isolates. Sequencing of a PCR-amplified fragment confirmed >99% homology with the sequence obtained for the LGA251 strain. Molecular typing of both isolates showed that they harbored an *agr* allele 3, were *spa* type t843, and belonged to clonal complex 130 (sequence type [ST] 130 for the cow isolate and ST1945, a single-locus variant of ST130 that differs by only 1 nucleotide within the *pta* gene, for the human isolate). These characteristics matched those of the most prevalent clones described by García-Álvarez et al. (*3*).

These isolates were also subjected to DNA microarray analysis by using the StaphyType Kit (Alere Technologies GmbH, Jena, Germany). Results confirmed assignment of the isolates to clonal complex 130, and showed that both isolates had hybridization profiles identical with those of 2 *mec*A variant isolates described by Shore et al. (*2*), except that the isolates were negative for *ccr*-A3 and *ccr*-B3. Both isolates were misidentified as methicillin-susceptible *S.* *aureus* by use of this microarray genotyping approach and the real-time PCR (GeneOhm Staph SR; BD Diagnostics, San Diego, CA, USA) because of *mec*A was not amplified.

The human isolate was determined to be methicillin sensitive by using the BD Phoenix PMIC/ID60 panel (BD Diagnostics) (oxacillin MIC 0.5 mg/L, cefoxitin MIC 4 mg/L, moxalactam MIC 16 mg/L). In addition to the inability of the Xpert MRSA/SA SSTI assay (Cepheid, Sunnyvale, CA, USA) reported by Shore et al. (*2*) to detect *mec*A variant isolates, these data confirm the inability of commercial molecular methods and some phenotypic panels currently available to identify all *mec*A variant isolates. These findings raise fears that such MRSA isolates are misidentified and that their prevalence is underestimated.

## Conclusions

Our results provide information on global distribution of the *mec*A variant gene. Geographic and temporal diversity of the isolates suggest that such strains are widely distributed and were not recently introduced in France. Moreover, animal cases were described only in the United Kingdom, and no cluster of clinical cases (2 cases of cow mastitis) was reported. Our data demonstrate the ability of such strains to cause clustered cases.

This new methicillin-resistance mechanism in *S. aureus* may be a new public health threat. Global dissemination of *mec*A_LGA251_ *S. aureus* should be investigated and controlled in humans and animals. Control measures should include rational use of antimicrobial drugs, accurate and rapid microbiological laboratory services, and specific infection-control measures.

In addition, the epidemiologic situation should be carefully monitored. However, such monitoring is made difficult by a combination of 3 issues. The first issue is the inability to detect *mec*A variant MRSA by using commercial (used for screening) molecular approaches or in-house (used for confirmation) amplification tests. The second issue is lack of sensitivity of some commercial phenotypic panels used for routine drug susceptibility testing. The third issue is the need for specific and easy-to-read phenotypic tests to confirm methicillin resistance caused by additional PBP (i.e., absence of synergy between oxacillin and amoxicillin/clavulanic acid disks to exclude borderline oxacillin-resistant *S. aureus* without being able to exclude a modified PBP-resistant *S. aureus* phenotype). The first step in overcoming this difficulty would be inclusion in the surveillance system systematic characterization of clinical strains harboring methicillin-resistance genes associated with susceptibility to all other antimicrobial drugs, a profile typical of *mec*A-variant MRSA isolates.

This report of new *mec*A variants in France confirms their wide geographic range, but many questions remain. The prevalence of *mec*A_LGA251_–positive isolates in France and other countries should be evaluated in livestock and humans. The origin, evolutionary mechanisms, potential animal reservoirs, and mode of dissemination of *mec*A-variant clones over large areas remain unknown. The clinical effect of expression of the PBP2a variant has not been definitively established in patients and should be explored in animal models.
